# Prevalence of hypertension and cross-sectional associations between household air pollution exposure and blood pressure among children in rural Rwanda

**DOI:** 10.1088/2752-5309/ae8ff9

**Published:** 2026-08-05

**Authors:** Christian Sewor, Ky Tanner, Vincent Cleveland, Bonnie N Young, Christian L’Orange, Rebecca Witinok-Huber, John Balmes, Egide Kalisa, Lindsay Zurba, Theoneste Ntakirutimana, Kayleigh P Keller, Howard H Chang, John Volckens, Maggie L Clark

**Affiliations:** 1 Department of Environmental and Radiological Health Sciences, Colorado State University, Fort Collins, CO, United States of America; 2 Department of Mechanical Engineering, Colorado State University, Fort Collins, CO, United States of America; 3 School of Public Health, University of California, Berkeley, Berkeley, CA, United States of America; 4 Department of Medicine, University of California, San Francisco, San Francisco, CA, United States of America; 5 Department of Epidemiology and Biostatistics, Western University, London, Ontario, Canada; 6 Education for Health Africa, Durban, South Africa; 7 Department of Environmental Health Sciences, School of Public Health, College of Medicine and Health Sciences (CMHS), University of Rwanda, Kigali, Rwanda; 8 Department of Statistics, Colorado State University, Fort Collins, CO, United States of America; 9 Department of Biostatistics and Bioinformatics, Rollins School of Public Health, Emory University, Atlanta, GA, United States of America

**Keywords:** particulate matter, black carbon, systolic blood pressure, diastolic blood pressure, cardiovascular

## Abstract

Household air pollution (HAP) contributes to the global cardiovascular disease burden. Early life exposures may impact future disease risk; however, evidence of HAP’s impact on blood pressure (BP) among children in low-resource settings is limited. We assessed baseline cross-sectional associations between personal exposure to fine particulate matter (PM_2.5_) and black carbon (BC) and children’s (8–15 years) systolic and diastolic BP (SBP, DBP) from the Sustainable Household Energy Adoption in Rwanda study. We enrolled 626 households in rural Eastern Rwanda that used traditional biomass fuels for cooking. Children wore Ultrasonic Personal Aerosol Samplers for 48 h monitoring of personal exposure, followed by BP measurement using standard protocols. Multiple linear regression was used to characterize associations between PM_2.5_ and BC exposure and BP in separate models; effect modification by age and sex was evaluated. Among 622 children (mean age = 11.8 years, standard deviation [SD] = 2.1; males = 303, females = 319), the average SBP and DBP were 105.8 mmHg (SD = 10.0) and 68.3 mmHg (SD = 7.6), respectively. BP percentiles were higher than those in a US reference population (e.g. 59.0% and 85.5% had SBP and DBP percentiles >50th, respectively; 24% were classified as having elevated BP and/or were hypertensive, defined as ⩾90th percentile). The median 48 h PM_2.5_ and BC concentrations were 192.9 *µ*g m^−3^ (25th percentile [*Q*1] = 119.7, 75th percentile [*Q*3] = 336.0) and 9.85 *µ*g m^−3^ (*Q*1 = 6.56, *Q*3 = 13.23), respectively. We did not observe evidence of associations between HAP and BP levels (e.g. PM_2.5_–SBP = 0.51 mmHg per interquartile range [IQR, 211.7 *μ*g m^−3^] increase, 95% confidence interval: −0.46, 1.49). We did not observe clear evidence of effect modification. Exposures well above international health-based guidelines may have limited our ability to observe associations if the true exposure–response is flat in that part of the global exposure continuum. Importantly, our BP data suggest an elevated cardiovascular disease burden among children in this low-resource setting compared to in the US, demonstrating a need for more research and the development of more appropriate reference data in these settings. ClinicalTrials.gov Identifier: NCT05668624.

## 1. Introduction

Household air pollution (HAP) from burning biomass fuels for domestic energy is a major contributor to the global disease burden, particularly for cardiovascular morbidity and mortality (Bennitt *et al* 2025). Worldwide, HAP affects more than 2 billion people, mostly in low and middle-income countries (LMICs) (Health Effects Institute 2025b). Exposure to HAP is especially high in Sub-Saharan Africa. In countries such as Rwanda, where this study was conducted, about 90% of the population relies on solid fuels for cooking (Health Effects Institute 2025a). In LMICs, in general, women and children have been reported to be disproportionately exposed to higher levels of HAP due to household tasks, such as fire starting, fire tending, and cooking (Chowdhury *et al* 2023, Zajac *et al* (the Council on Environmental Health and Climate Change) 2025).

Exposure to air pollution can induce oxidative stress, systemic inflammation, and endothelial dysfunction, and can also affect the autonomic nervous system, leading to higher sympathetic and lower parasympathetic tone (Brook *et al* 2010, Lee *et al* 2015, Newby *et al* 2015, Lederer *et al* 2021). These intermediate states lie along biological pathways that contribute to adverse cardiovascular health outcomes, including high blood pressure (BP) (Seaton *et al* 1995, Perez *et al* 2015, Miller 2020). Children are often more susceptible to the health impacts of air pollution because of their developing respiratory systems, increased exposure from higher breathing rates, and behavioral tendencies (Chong-Neto and Filho 2025). The largest evidence base comes from studies of ambient fine particulate matter (PM_2.5_); meta-analyses, which primarily included studies from high-and upper-middle-income countries, suggest positive associations between exposure and BP levels among younger children and adolescents for both short-term and long-term PM exposure (Huang *et al* 2021, Yan *et al* 2021, Liu *et al* 2023, Tandon *et al* 2023).

In the case of HAP, exposure–response analyses from interventional studies and other observational studies conducted across adult and child populations (Baumgartner *et al* 2012, 2018, Burroughs Pena *et al* 2015, Quinn *et al* 2016, Nicolaou *et al* 2022, Ye *et al* 2022a, 2022b, Bates *et al* 2025), although inconsistent, have suggested that higher HAP exposure is associated with higher BP. The evidence, however, for children is minimal, with only one study, to the best of our knowledge, exploring this association (Baumgartner *et al* 2012). That study, which was conducted among Chinese school children between the ages of 5 and 14 years (Baumgartner *et al* 2012), did not observe an association between either PM_2.5_ or black carbon (BC) levels on systolic BP (SBP) and diastolic BP (DBP)

Considering that early-life environmental exposures, such as HAP, can impact future disease risk (Chen and Wang 2008), understanding how HAP affects early-life cardiovascular health among children living in LMICs, where the burden of HAP is greatest (Bennitt *et al* 2025), and the evidence is least, can be crucial in mitigating future cardiovascular disease burden in these regions (Yang *et al* 2020; Urbina *et al* 2023). This is particularly important, as children within these settings have been reported to be exposed to PM_2.5_ concentrations that were 14% and 100% higher than those of their mothers and fathers, respectively (Tanner *et al* 2025).

This study bridges an important research gap by leveraging baseline cross-sectional data from a randomized controlled trial to assess the association between PM_2.5_ and BC exposure and BP among children (8–15 years) in rural Rwanda.

## 2. Methods

### 2.1. Study design and population

The Sustainable Household Energy Adoption in Rwanda (SHEAR) study was a randomized intervention of liquefied petroleum gas for cooking and solar electricity for lighting.

The study was conducted in the Karambi and Isangano communities within the Ndego District of the Eastern Province of Rwanda. These communities are primarily agrarian, with most households residing in homes constructed from mud bricks and with metal roofs and using traditional stoves that relied mainly on biomass fuels for cooking (Tanner *et al* 2025).

In SHEAR, households were included if they used biomass as the primary cooking fuel, were not involved in commercial cooking, had adult male or female heads of household (18+ years of age) willing to consent, and at least one child (aged 8–15 years) willing to assent, there was no smoking in the household, had no plans of moving out of the study site within a 3 year timeframe, and for adult females, they were not currently pregnant.

In total, 626 households were recruited, with data collected for a child and at least one adult.

All children provided informed assent with parental consent before participating in the study. This research received approval from the Rwanda National Ethics Committee (No. 00001497) and the Institutional Review Board at Colorado State University (CSU) (IRB No. 3946).

### 2.2. Exposure assessment

We have described the exposure assessment method used in this trial in detail in a previous publication (Tanner *et al* 2025). Briefly, the exposures of interest, 48 h-averaged PM_2.5_ and BC, were collected via the Ultrasonic Personal Aerosol Samplers (UPAS). Participants wore a UPAS near their breathing zone for a continuous 48 h period while going about their daily routines and placed the UPAS on an external battery charger overnight while sleeping. The UPAS is a wearable air quality monitor that uses a quiet piezoelectric pump to draw air at 1 l min^−1^ through a 2.5 *μ*m size-selective cyclone inlet and onto a 37 mm PTFE sampling filter (PT37 MTL LLC, Minneapolis, Minnesota) (Leith *et al* 2020). A separate, miniature fan draws air into a light-scattering sensor (SPS30, Sensirion AG, Stäfa, Switzerland) at the front of the unit to estimate PM_2.5_ concentration at 30 s intervals (Sensirion 2025). In addition, the UPAS records GPS, accelerometer, temperature, relative humidity, and atmospheric pressure data at the same 30 s intervals.

Personal air sampling filters were pre- and post-weighed in triplicate (XS3DU, Mettler-Toledo, Columbus, Ohio, USA) in a climate-controlled environment using an automated robotic process at CSU (L’Orange *et al* 2021). Post-sampling filters were stored at −80 °C upon receipt by CSU. Filter field blanks were collected at the beginning of each sampling day and used to account for potential measurement artifacts arising from shipping and handling.

Gravimetric PM_2.5_ concentrations were calculated by dividing the blank-corrected filter mass, the difference between the post- and pre-sampling measurements subtracted by the monthly average difference between post- and pre-deployment field blanks, by the volume of air sampled through the pump over the measurement period (Tanner *et al* 2025). The PM_2.5_ concentration limit of detection (LOD) was estimated for each month using the formula: LOD = *μ_b_* + 3σ*_b_* (*μ*_b_ is the monthly mean blank filter loading, and *σ*_b_ is the monthly blank filter loading SD). Samples with a value below the LOD were substituted with the LOD/√2 (Hewett and Ganser 2007).

To estimate the final 48 h-averaged PM_2.5_ concentrations, a unique correction ratio was computed for each sample by dividing the sample average real-time reported PM_2.5_ concentration (30 s observations for the times the filter pump was active) by the gravimetrically derived average concentration. The inverse of this correction ratio was multiplied by each 30 s PM_2.5_ concentration estimate for each corresponding sample. This provided us with a more complete data set than if we used the gravimetry-derived concentrations alone, since the real-time PM_2.5_ sensor was active even if the filter pump had to shut off or was duty cycled due to low battery. These gravimetric-corrected 30 s PM_2.5_ concentrations were used to estimate the 48 h-averaged PM_2.5_ concentrations reported (Tanner *et al* 2025).

BC mass concentrations were calculated from the change in 880 nm light transmission through each filter before and after sampling with the Sootscan Transmissometer (OT21, Magee Scientific) (L’Orange *et al* 2021). Calculations used a filter effective diameter of ∼31 mm, filter-specific sample volumes, and a mass absorption cross-section of 9.0 m^2^g^−1^ (Presler-Jur *et al* 2017).

### 2.3. Outcome assessment

SBP and DBP measurements followed procedures developed by the American Heart Association and adopted in the National Health and Nutrition Examination Survey (Muntner *et al* 2019, CDC 2021). Participants avoided eating, drinking alcohol or caffeine (e.g. coffee, tea, Coke), cooking, smoking, exercising, and bathing for 30 min prior to BP measurement. SBP and DBP readings were taken after the subject had been seated and rested for 5 min using a validated portable cuff device (Omron HEM 711AC) (Omron 2021), placed on their upper right arm, which was supported at a 90° angle. Different cuff sizes were used based on the participant’s arm size, according to the manufacturer (Omron 2021). Participants were instructed to sit with their backs supported, legs uncrossed, and feet on the floor or on a stool if their feet did not reach the floor. Initially, three consecutive readings were taken at 2 min intervals. If the absolute difference between the second and third SBP readings was not within the range of 4 mmHg, a fourth reading was taken. This was done to improve the precision of the BP readings, as recommended by the American Heart Association (Pickering *et al* 2005). The average BP (both SBP and DBP) was then calculated from the pair of readings with the smallest difference among the second, third, and fourth readings.

Percentiles of age-, sex-, and height-adjusted BP were estimated for each child based on the National Institutes of Health/Center for Disease Control guidelines and using the *pedbp* package in *R* (US Department of Health and Human Services 2004, Martin *et al* 2022). We chose this guideline because child nomograms are unavailable in Africa (Craig *et al* 2023), enabling comparison of BP percentiles for children in this population with those of children born in the United States. To assess the burden of high BP among children, we categorized BP percentiles into elevated SBP/DBP (90th-< 95th percentile) and hypertension (⩾95th percentile) per NIH guidelines (Benenson *et al* 2020).

### 2.4. Covariates

The study questionnaire was administered by a trained local study team using the REDCap electronic data capture tools hosted at the University of Colorado (Harris *et al* 2009). The questionnaires were administered in Kinyarwanda by a team member who was fluent in the local dialect. Data on a wide array of demographic (e.g. age and sex) and socio-economic information were self-reported by the study participants. Participants’ ages were based on self-reported birth dates; the year of birth was confirmed with a government-issued identification.

Children’s height was measured using a standardized portable stadiometer (SECA 213, seca GmbH &Co Hamburg, Germany). The field team set up a stadiometer in participants’ homes, asked them to remove their shoes and ornaments, and then stand against the device. After three breaths and straightening, their height was measured to 0.1 cm. This process was repeated three times, and the average height was recorded in REDCap.

The weighted asset index was calculated based on data provided on the presence and absence of a list of household items (household assets: mobile phone, smartphone [if ownership of a mobile phone], radio, television, bank account, community savings group, bicycle, motorcycle/scooter, car/truck) collected via the study questionnaire. This variable was estimated by ranking the nine household material items by prevalence (i.e. the least prevalent item in the study population received a score of 9, while the most prevalent item received a score of 1), then the ranked scores were summed for each participant to generate a composite score (Howe *et al* 2012).

We obtained information on the time of BP measurement and categorized it as morning, noon, or evening.

### 2.5. Statistical analysis

The characteristics of the study population were presented as counts/percentages for categorical variables and means with SD or medians with the first (Q1) and third quartiles (*Q*3) for continuous variables. Boxplots were used to visualize the distribution of the continuous exposure and outcome variables within this population. Spearman’s rank correlation was used to explore the correlation between PM_2.5_ and BC.

Linear regression models assessed the association between PM_2.5_ and BC concentrations and average SBP and DBP levels (mmHg) among the children. We adjusted for the following covariates identified *a priori,* in line with a directed acyclic graph (figure S1). The model was adjusted for age (years), weighted asset index scores, time of day at BP measurement (morning, noon, and evening), and sex (male and female), whilst height (cm) was included to improve precision, not because it was regarded as a confounder. We reported model estimates as changes in SBP and DBP in mmHg per interquartile range (IQR) increase in PM_2.5_ and BC concentrations.

The primary model included all participants with BP data and at least 36 h of exposure measurement. One child was excluded due to missing height data.

We explored effect modification by age and sex by fitting models with two-way (PM_2.5_ or BC and sex) and three-way (PM_2.5_ or BC, sex, and age group [<13 years and ⩾13 years]) interactions. Given the potential impact of puberty on cardiovascular health, we chose a somewhat conservative age cutoff of 13 years based on the reported age at menarche among African children (13.82; 95% CI: 13.08, 14.55) from the meta-analysis by Moodie *et al* (2020).

Several sensitivity analyses were conducted. We evaluated the associations among participants with precise SBP and DBP (participants with average BP-based readings within 4 mmHg) as well as among participants who had both valid BC and valid PM_2.5_ readings (i.e. to ensure any potential differences in effect estimates were not based on the inclusion of different sub-populations). We also explored potential non-linearity in the exposure–response relationships by modeling the exposures as natural cubic spline terms, with 5 degrees of freedom. In a three-way interaction model involving PM_2.5_ or BC, sex, and age, we modeled age as a continuous variable using a spline term (3 degrees of freedom), allowing the linear relationship between exposures and the outcome with respect to sex to vary non-linearly across age. This allowed us to explore the potential impact of choosing to dichotomize age in our primary 3-way interaction models. We also conducted an additional analysis only amongst participants who were deemed compliant with regard to wearing the exposure monitoring equipment. This compliance measure, based on UPAS accelerometer data, was used to identify participants who exhibited movement consistent with wearing the UPAS for at least 75% of waking hours (6:00–21:00) (Tanner *et al* 2025).

Finally, missing data occurred for various reasons, which may raise concerns about selection bias. To evaluate potential selection bias from missingness, we compared the distribution of covariates among participants included in the final analysis and those excluded. All analyses were run on R version 4.4.3 (R Core Team 2025).

## 3. Results

A flow diagram of the total number of children included in the analyses and the reasons for missing data and exclusions is shown in figure S2 (A) and (B). Of the 626 initially recruited, 504 (80.5%) and 413 (66.0%) were included in the primary analysis for PM_2.5_ and BC, respectively. The main reason for missingness was related to quality control of exposure measurements.

**Figure 1.**
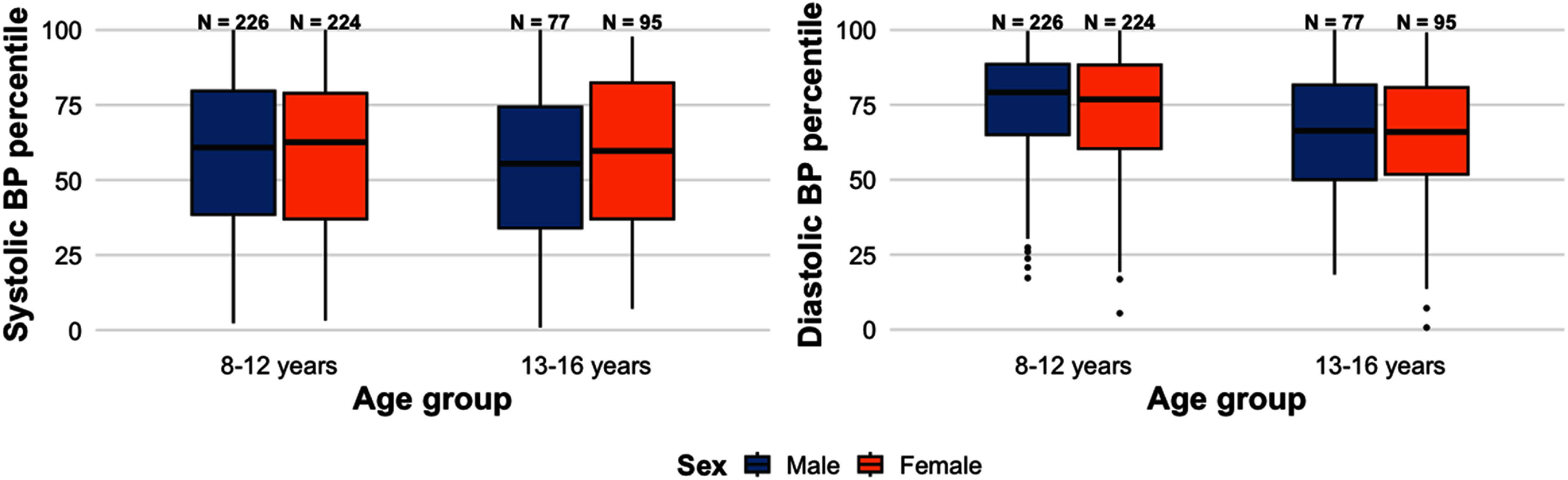
Distribution of blood pressure percentiles by participant age and sex. Percentiles are calculated relative to a representative sample of US children (US Department of Health and Human Services 2004, Martin *et al* 2022).

**Figure 2.**
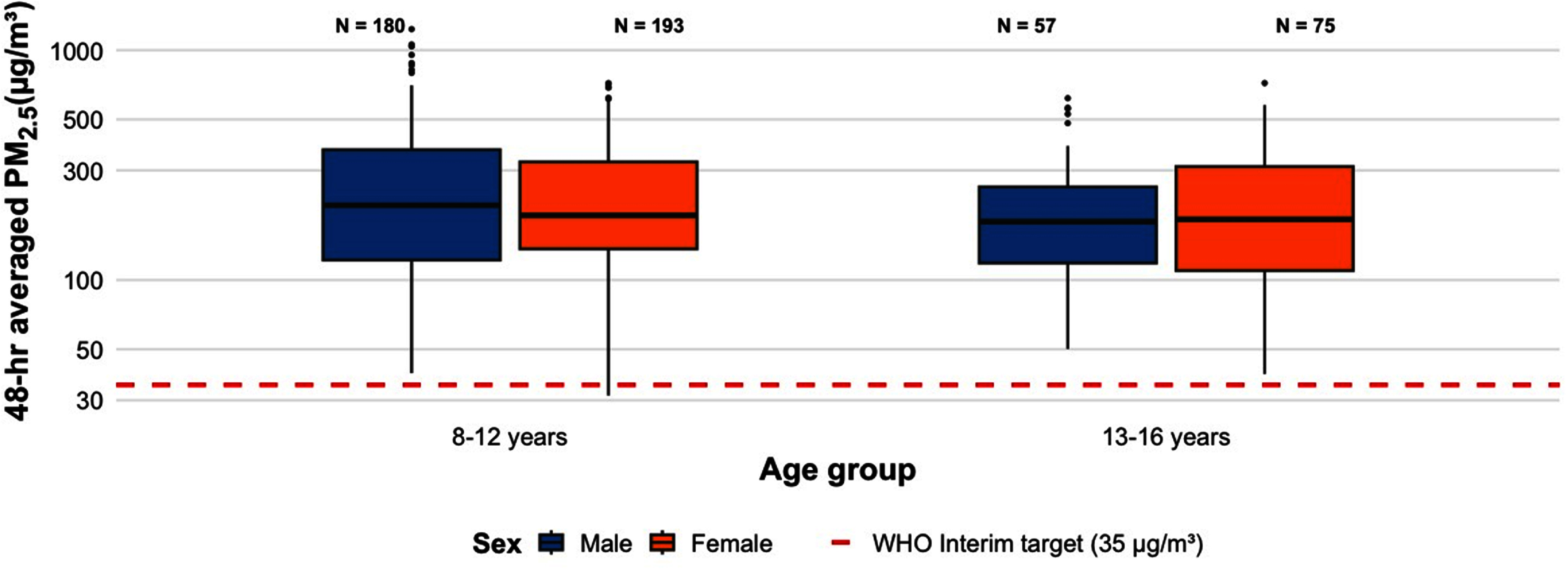
Distribution of 48 h personal PM_2.5_ across age and sex. The *y*-axis is on the log scale, and the outliers are defined as points at least 1.5⋅IQR beyond the box limits.

**Figure 3.**
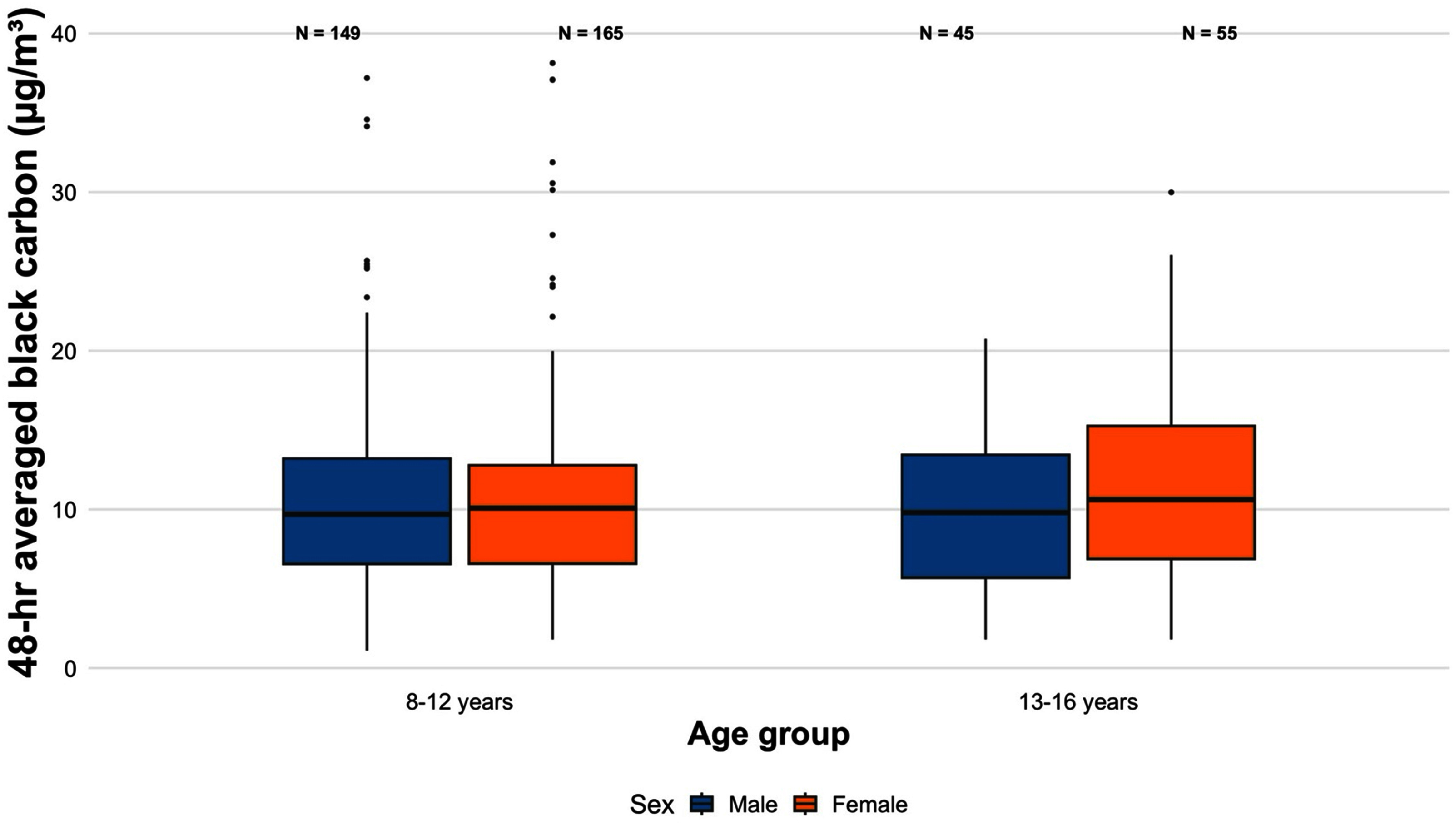
Distribution of 48 h personal PM_2.5_ and black carbon exposures by participant age and sex. Outliers are defined as points at least 1.5⋅IQR beyond the box limits.

The characteristics of the study population are presented in table 1 and figures 1–3. Of the 622 children, 303 were male, and 319 were female. The mean age and weighted asset index score were 11.8 years (SD = 2.1) and 7.2 (SD = 5.3), respectively. There was no difference in mean age across sex, but male children lived in households with a higher weighted asset index score. The average height was 141 cm (SD = 11.9), with female children being slightly taller. The average SBP and DBP within the population were 105.8 mmHg (SD = 10.0) and 68.3 mmHg (SD = 7.6), with the average SBP being slightly higher among female children, although DBP was similar across sex. Female children had a slightly higher SBP percentile (Female = 58.0 [SD = 25.9] versus Male = 55.8 [SD = 25.9]), whereas male children had a slightly higher DBP percentile (Male = 71.9 [SD = 18.9] versus Female = 70.1 [SD = 20.1]). However, distribution of BP percentiles was similar across age groups and sex (figure 1). About 24% of the children had an elevated BP or were defined as hypertensive based on their BP percentiles. Median 48 h averaged PM_2.5_ and BC concentrations were 192.9 *µ*g m^−3^ (*N* = 505, 25th percentile= 119.7, 75th percentile= 336.0) and 9.85 *µ*g m^−3^ (*N* = 413, 25th percentile = 6.56, 75th percentile = 13.23), respectively, with no difference in levels among male and female children (figures 2 and 3). PM_2.5_ and BC were moderately correlated (*ρ*= 0.59) (figure S3).

**Table 1.** Study characteristics of children.

Characteristics	*N*	*N* (%)/Mean (SD)/ Median (*Q*1, *Q*3)	Male (*N* = 303)	Female (*N* = 319)
Age (years)	622	11.8 (2.1)	11.6 (2.1)	11.9 (2.2)
Weighted asset index	621	7.2 (5.3)	7.6 (5.6)	6.8 (5.0)
Height (cm)	620	141.1 (11.9)	139.7 (11.8)	142.5 (11.9)
Systolic blood pressure (mmHg)	622	105.8 (10.0)	105.0 (10.0)	106.6 (9.8)
Systolic blood pressure percentile	622	56.9 (25.9)	55.8 (25.9)	58.0 (25.9)
Diastolic blood pressure (mmHg)	622	68.3 (7.6)	68.2 (7.7)	68.4 (7.6)
Diastolic blood pressure percentile	622	71.0 (19.6)	71.9 (18.9)	70.1 (20.1)
Proportion with elevated BP/Hypertension (⩾90th percentile)	622	147 (23.6%)	72 (23.8%)	75 (23.5%)
Proportion with elevated BP (90th–< 95th percentile)	622	65 (10.5%)	37 (12.2%)	28 (8.8%)
Proportion with Hypertension (⩾95th percentile)	622	82 (13.2%)	35 (11.6%)	47 (14.7%)

*Time of BP measurement*				

Morning		267 (42.9%)	129 (42.6%)	138 (43.3%)
Noon		344 (55.3%)	171 (56.4%)	173 (54.2%)
Evening		11 (1.8%)	3 (1.0%)	8 (2.5%)
Precise systolic measurement	622	592 (95.2%)	293 (96.7%)	299 (93.7%)
Precise diastolic measurement	622	456 (73.3%)	226 (74.6%)	230 (72.1%)
48-hr averaged PM_2.5_ (*μ*g m^−3^)	505 (Male = 237/ Female = 268)	195.3 (124.9, 338.7)	202.0 (122.1357.5)	191.0 (126.4, 323.4)
Number with PM_2.5_ above WHO- Annual IT-1 (35 *μ*g m^−3^)	505 (Male = 237/ Female = 268)	503 (99.6%)	237 (100.0%)	266 (99.3%)
48-hr averaged black carbon (*μ*g m^−3^)	414 (Male = 194/ Female = 220)	9.85 (6.56, 13.23)	9.72 (6.50, 13.37)	10.30 (6.59, 13.19)

BP = Blood pressure, N = number of observations, PM_2.5_ = fine particulate matter ⩽2.5 *µ*ms in diameter, SD = Standard deviation, *Q*1 = First quartile/25th percentile, *Q*3 = Third quartile/75th percentile, WHO- IT-1- World Health Organization Annual-Interim Target-1.

**Figure 4.**
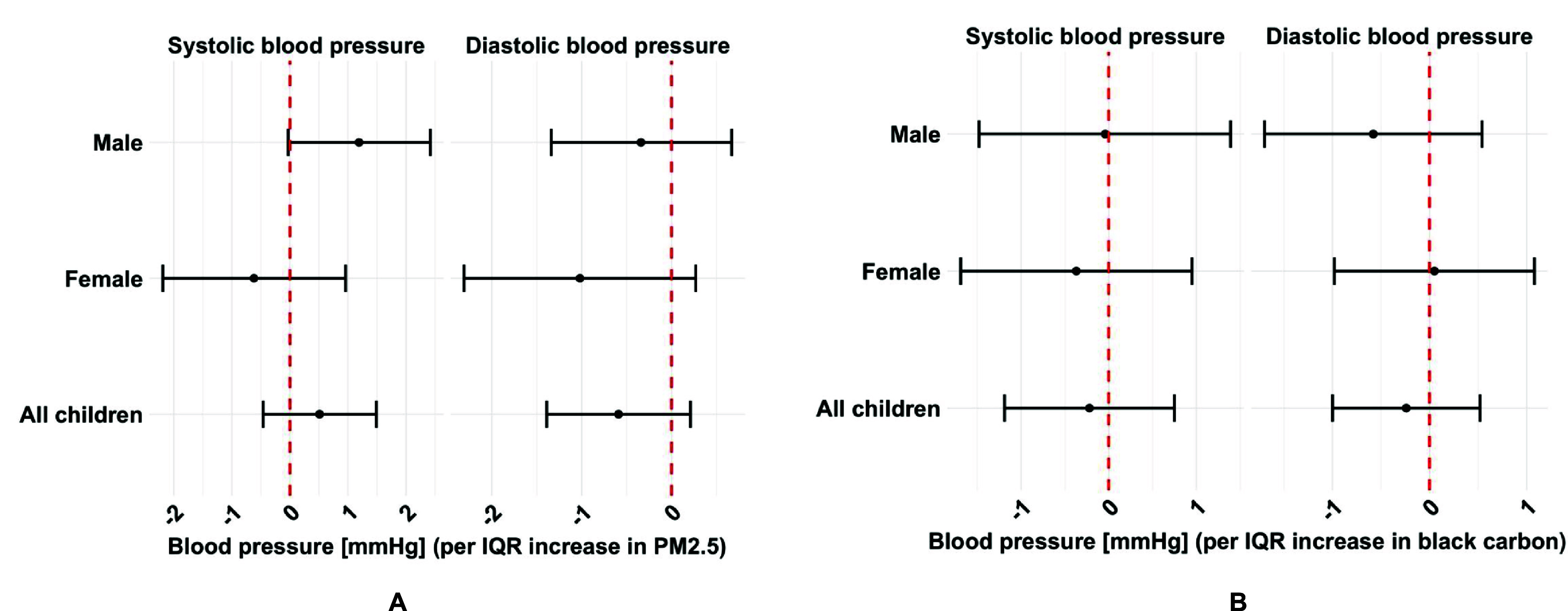
Association between 48 h HAP exposure and child blood pressure levels, (**A)**–PM_2.5_ results; (**B)**–BC results). PM_2.5_ Interquartile range= 211.7 *μ*g m^−3^; BC Interquartile range= 6.7 *μ*g m^−3^; Models were adjusted for weighted asset index, age (years), height (cm), sex, and time of day of BP measurement, PM_2.5_ N: All children= 483, Male = 226, Female= 257, *p*-interaction by sex = 0.07 for SBP and 0.41 for DBP; BC N: All children= 413, Male = 194, Female= 219, *p*-interaction by sex = 0.74 for SBP and 0.41 for DBP.

Results on the association between PM_2.5_ and BC with SBP and DBP are presented in figure 4 and tables S1 and S2. We did not observe an overall association between PM_2.5_ exposure and SBP (*β*= 0.51 mmHg per IQR increase, 95% CI: −0.46, 1.49) and DBP (*β*= −0.59 mmHg per IQR increase, 95% CI: −1.39, 0.21).

In the two-way interaction analysis (PM_2.5_ and sex), we observed that among male children, an IQR higher PM_2.5_ exposure was associated with 1.19 mmHg higher SBP levels (95% CI: −0.03, 2.42); among female children we did not observe an association (*p*-for interaction= 0.07). We did not observe evidence that the association between PM_2.5_ and DBP differed by sex (*p*-for-interaction= 0.41).

In the three-way interaction (figure S4) involving PM_2.5_, sex, and age group, among younger female children (8–12 years), an IQR higher PM_2.5_ exposure was associated with 1.61 mmHg lower DBP levels (95% CI: −3.10, −0.13), albeit the *p*-for-interaction values were consistent with the null for both DBP and SBP.

For BC analysis, we did not observe an association for SBP (*β*= −0.22 mmHg per IQR increase, 95% CI: −1.19, 0.75) or DBP (*β*= −0.24 per IQR increase, 95% CI: −1.00, 0.52). In the two-way (involving PM_2.5_ and sex) and three-way interactions (figure S5) (involving PM_2.5_, sex, and age group), we did not observe evidence of effect modification (Two-way: SBP *p*-for-interaction = 0.74; DBP *p*-for-interaction = 0.41; Three-way: SBP *p*-for-interaction = 0.54; DBP *p*-for-interaction = 0.93).

Overall, we observed similar patterns of association across all sensitivity analyses for PM and BC-related analyses (tables S1 and S2). Results exploring the shape of the associations in a non-linear fashion (i.e. modeling exposure as splines and modeling age as a spline in the three-way interaction) showed similar patterns to those in the primary exposure–response analyses: an overall suggestive positive pattern with respect to SBP and a negative pattern with respect to DBP; albeit these associations were consistent with the null (figures S6–S11).

Also, the distribution of key variables among participants included in the final analysis versus those excluded was similar (tables S3 and S4).

## 4. Discussion

In this study of a population of children with relatively high exposure to HAP and relatively high prevalence of elevated BP, we did not observe evidence of an overall association between PM_2.5_ and BC concentrations and SBP and DBP levels. In the PM_2.5_ analyses, we observed some inconsistent patterns of associations across subgroups of sex and age, but all *p*-for-interaction values were >0.05. None of the BC-related analyses indicated evidence of effect modification.

Despite these findings, the distribution of BP warrants further consideration. Compared with the regional Sub-Saharan African estimates reported in a meta-analysis which focused on adolescent-aged children (10–19 years) (Chen *et al* 2023), the reported average SBP (111 mmHg, 95% CI: 108, 114) was slightly higher than the observed estimate in this study (105.8 mmHg), while their DBP estimate (68 mmHg, 95% CI: 66, 70) was similar to ours (68.3 mmHg). In this meta-analysis, the reported pooled prevalence for hypertension (the authors used the term elevated BP, but their definition coincides with our definition for hypertension; 95th percentile threshold or higher for participants younger than 13 years, and an absolute threshold of 120/80 mmHg or higher for those aged 13–19 years) in Sub-Saharan Africa of 9.9% (95% CI: 7.3, 12.5) and in East Africa of 7.4% (95% CI: 4.5, 10.4)) was lower than the prevalence of hypertension (⩾95th percentile) of 13.2% reported in our study. The sex-specific prevalences were, however, different, with 13.4% (95% CI 12.9, 13.9) and 11.9% (11.3, 12.4) reported for males and females, respectively in the meta-analysis (Chen *et al* 2023), whereas 11.6% and 14.7% were observed in our study from males and females, respectively.

Similar meta-analyses conducted across Africa, which focused on children within wider age ranges (2–19 years (Noubiap *et al* 2017) and 3–19 years (Crouch *et al* 2022)), reported prevalence estimates for hypertension that were slightly lower than those reported in our study population. The only Rwandan study, to the best of our knowledge, that assessed the burden of child hypertension was a cross-sectional study conducted among 3 year-old children. It also reported a high prevalence of elevated BP/hypertensive (40.4%) (Ndagijimana *et al* 2023).

Taken together, these previous studies alongside ours highlight a high prevalence of undiagnosed child hypertension and the sparsity of local evidence, both of which are serious public health concerns. This is particularly important because early-life cardiovascular health, while having its own consequences on early cardiovascular disease markers such as arterial stiffness parameters and left ventricular mass index (Raina *et al* 2022), is likely to impact cardiovascular disease risk in adulthood (Chen and Wang 2008, Scott *et al* 2025). As reported in a 2020 systematic review by Yang *et al* (2020), elevated BP in childhood or adolescence was associated with increased odds of poor cardiovascular outcomes in adulthood (high pulse wave velocity OR = 1.83, 95% CI, 1.39, 2.40; high carotid intima-media thickness OR= 1.60, 95% CI: 1.29, 2.00, and left ventricular hypertrophy OR = 1.40, 95% CI: 1.20, 1.64).

Additionally, the high BP percentiles in this population, relative to a US reference population, raise concerns about differences in BP distribution across populations. Although the BP percentiles used in this study were standardized to reflect the demographic profile of the US, which includes children of African ancestry, the lack of child BP nomograms in Africa means that BP percentiles are typically categorized using international guidelines that lack data on children residing in African countries (Craig *et al* 2023).

Very few studies have explored how HAP impacts children’s BP (Baumgartner *et al* 2012, Daouda *et al* 2024), with only one study, to the best of our knowledge, that included children aged 5–14 years old (Baumgartner *et al* 2012). In this cross-sectional study highlighted earlier, the authors did not observe evidence of an association between HAP exposure (PM_2.5_ and BC) levels and SBP and DBP. The authors noted that the null findings could have been due to the presence of residual confounding, as well as the reported relatively low exposure levels (Mean (SD)= 58 *µ*g m^−3^ (47), Range= 14–393 *μ*g m^−3^) observed among the children (Baumgartner *et al* 2012). In our study, however, because almost all the children were exposed to very high HAP levels as compared to WHO- Annual IT-1 (35 *μ*g m^−3^), we think it is likely that associations we explored in this population are occurring at the upper tail of the hypothesized exposure–response curve, where any additional increase in exposure often exerts little to no effect (Baumgartner *et al* 2011). While it is difficult to confirm this due to the lack of exposure–response curves on HAP exposures and children’s BP, evidence from the adult population suggests these relationships are often non-linear (Baumgartner *et al* 2011).

The mostly null results observed in the case of HAP differ from suggestive positive associations observed in the ambient pollution literature (Chowdhury *et al* 2023), although it is worth noting that a recent cross-sectional study conducted among Ghanaian school children (7–14 years) reported mixed findings (Alli *et al* 2026). While Alli *et al* (2026) did not observe an association between residential and school PM_2.5_ levels and BP (SBP and DBP) they reported that residential and school BC and NO_2_ were negatively associated with DBP (Alli *et al* 2026). Because there are often substantial differences in the sources and compositions of ambient and HAP, it is difficult to directly compare these results with ours.

Our results from the effect modification analyses suggest potential sex differences in the association between HAP and BP (positive associations among male children). This association should be interpreted cautiously, however, and the result necessitates further evaluation of how these demographic characteristics may modify the impact of HAP on BP levels. Among published HAP studies, Daouda *et al* (2024) evaluated sex-specific differences among 4 year-old children. They observed a positive relationship between child CO exposure and DBP-*z*-score among male children but not among female children or with respect to SBP-*z*-score. Also, in an attempt to explore the potential impact of puberty, Baumgartner *et al* (2012), explored associations limited to girls ages 10–14 years old and boys ages 12–14 years old, but found similar null associations as observed in their primary analysis.

## 5. Strengths and limitations

While our study is not without limitations, we do not believe that issues of information or selection bias substantially affected our findings. In the case of the former, the robust exposure and outcome assessment methods employed in this study, coupled with the consistency of the association across various sensitivity analyses, which are obvious strengths of this study (i.e. leveraging additional exposure assessment compliance, leveraging precise outcome data as quality controls), make us reasonably confident that such bias was minimized. However, there may still be exposure misclassification due to measurement error, as a single time-point, 48 h-averaged measurement may not reflect typical long-term exposure, which was the goal of this study (Carroll *et al* 2006, Keller and Clark 2022). In such a case, the ensuing bias due to classical measurement error (Carroll *et al* 2006, Keller and Clark 2022) will likely be non-differential, thereby biasing the estimated change in BP towards the null.

In the case of selection bias, the primary source of missingness relates to the unavailability of exposure data. However, the distribution of key variables among participants included in the final analysis versus those excluded (tables S3 and S4) was similar. While residual confounding may still be present from SES-related factors, particularly dietary diversity, we anticipate that the ensuing bias would be upward (assuming that dietary diversity will be negatively associated with BP (Izadi *et al* 2020) and with HAP through SES). The magnitude of this bias will, however, be small, as adjusting for the weighted asset index will likely minimize some of the confounding due to this factor.

We relied on baseline cross-sectional data; hence, this analysis does not establish temporality. However, prospective analyses from the entire trial will ultimately address this issue and assess how longer-term exposures may affect BP over the study period. Also, it was difficult to assess how puberty may have modified the association between HAP and BP, since we did not collect hormonal data. While the age-stratified analyses offer some insight into this, more research is warranted surrounding methods to better understand and account for the precise timing of puberty, particularly in LMICs (Moodie *et al* 2020).

Overall, despite the limitations, the robust exposure and outcome assessment methods enabled us to collect high-quality data within this understudied population of children.

## 6. Conclusion

In this cross-sectional analysis, although we did not observe an overall association between PM_2.5_ and BC exposures and BP, our study findings contribute to the existing body of evidence on the growing burden of cardiovascular disease among children in low-resource, Sub-Saharan settings. We recommend conducting robust prospective studies to clarify how early-life exposure to HAP affects cardiovascular health over the lifespan.

## Data Availability

Individual-level data are not publicly available due to ethical and participant confidentiality restrictions. In accordance with NIH data-sharing policies, de-identified data and supporting documentation may be made available to qualified researchers upon reasonable request, subject to institutional approvals and a data use agreement. The R software version 4.4.3 (R Core Team 2025) used in this study is publicly available. The data cannot be made publicly available upon publication because they contain sensitive personal information. The data that support the findings of this study are available upon reasonable request from the authors. Supplementary data 1 available at: https://doi.org/10.1088/2752-5309/ae8ff9/data1.

## Disclaimer

The content of this work is solely the responsibility of the authors and does not necessarily represent the official views of the National Institutes of Health. The funders had no role in the study design. The authors declare the following competing financial interest(s): John Volckens is a scientific founder of Access Sensor Technologies LLC, which manufactures and sells the UPAS v2.1 PLUS (monitor used for data collection). The terms of this arrangement have been reviewed and approved by Colorado State University in accordance with its conflict-of-interest policies.
